# Progressive Multifocal Leukoencephalopathy Presenting as Primary CNS Malignancy in an Immunocompetent Patient

**DOI:** 10.7759/cureus.45815

**Published:** 2023-09-23

**Authors:** Rachel D Truong, Vamshi K Vadlapatla, Nicholas G Avgeropoulos

**Affiliations:** 1 Department of Internal Medicine, Orlando Regional Medical Center, Orlando, USA; 2 Department of Hematology and Medical Oncology, Orlando Regional Medical Center, Orlando, USA

**Keywords:** jc virus, malignancy, cns lymphoma, immunocompetent, pml, progressive multifocal leukoencephalopathy

## Abstract

Progressive multifocal leukoencephalopathy (PML) is an infection caused by the John Cunningham virus (JCV), usually in an immunocompromised host. We present the case of a 74-year-old male who presented with a six-week history of progressive memory loss, episodic confusion, and aphasia. Cranial nerve, motor, sensory, and coordination testing were unremarkable. Magnetic resonance imaging (MRI) of the brain and spectroscopy were concerning for primary CNS lymphoma vs. diffuse glioma. Microscopic examination after the patient underwent left frontal stereotactic brain biopsy was suggestive of a viral infection, and further testing with JCV DNA in-situ hybridization (ISH) confirmed the diagnosis of PML. The patient's condition started resolving without treatment. This case demonstrates, to our knowledge, the first known case of primary PML masquerading as CNS lymphoma in modern literature.

## Introduction

Progressive multifocal leukoencephalopathy (PML) is an often fatal and progressive demyelinating disease caused by the John Cunningham virus (JCV), which usually remains latent in immunocompetent hosts. Immunocompromised patients, those with known malignancies, and patients undergoing immunotherapy are at risk for reactivation of the virus and subsequent PML [1]. It is quite rare for a patient to present with primary PML in an immunocompetent state [2-11]. Because of this, the patient requires extensive diagnostic evaluation, leading to diagnostic brain biopsy. Here, we describe a case of primary PML presenting as central nervous system (CNS) lymphoma in an immunocompetent patient. 

## Case presentation

A previously active and healthy 74-year-old male with no history of chronic medical conditions or chronic medication use initially presented with six weeks of progressive memory loss, episodic confusion, and expressive aphasia. On evaluation, he was awake, alert, and oriented to time, place, and person. There were no issues with attention or concentration. He had partially accurate yes/no answering capacity, but his speech fluency was otherwise significantly impaired with a paucity of verbal speech aside from yes or no answers. He was able to follow one to three-step requests and had dramatically reduced immediate and short-term memory with an inability to communicate his degree of understanding. His long-term memory was similarly impaired. Due to his expressive aphasia, delayed recall was not able to be tested. He had no difficulty with visuospatial functions. Cranial nerve, motor, sensory, and coordination testing were normal.

**Figure 1:**
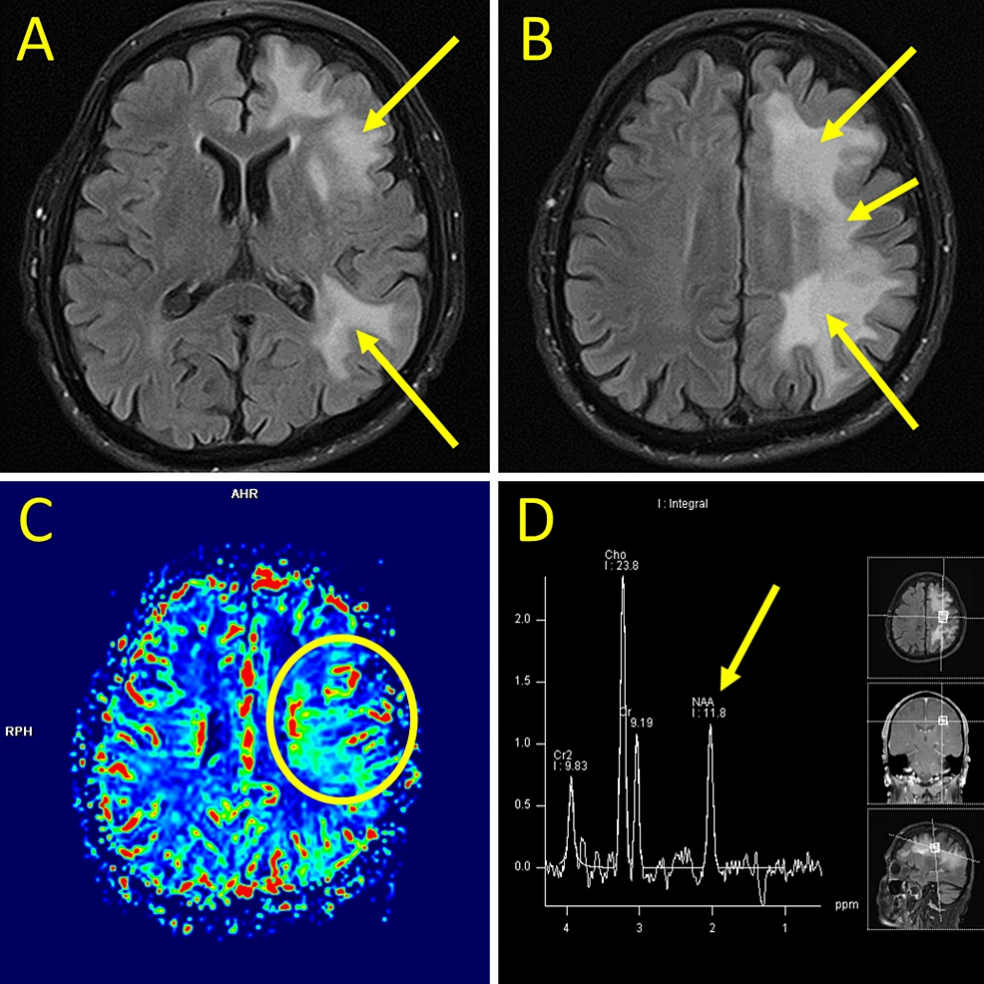
Key images from MRI brain with and without contrast, perfusion imaging, and MR spectroscopy (a) T2 flair post-contrast showing diffuse abnormal left-hemispheric signaling (yellow arrows). (b) T2 flair post-contrast showing progression of diffuse abnormal left-hemispheric signaling (yellow arrows). (c) MRI perfusion showing elevated cerebral blood volume at the multilobar left hemispheric area of involvement (yellow circle). (d) MR spectroscopy showing a decrease in N-acetyl aspartate (yellow arrow).

He underwent sequential magnetic resonance imaging (MRI) with and without contrast and perfusion of his brain, which revealed relatively diffuse abnormal signaling expanding the white matter of the left cerebral hemisphere, progressive T2 flair post-contrast (Figure 1A, 1B), and elevated cerebral blood volume at the multilobar left hemispheric area of involvement (Figure 1C). Findings were most pronounced at the left frontal operculum. MR spectroscopy showed a nonspecific decrease in N-acetyl aspartate (NAA) (Figure 1D).

In aggregate, these imaging and clinical findings were worrisome for malignant multifocal tumor activity, such as glioblastoma or primary CNS lymphoma. Screening for autoimmune, infectious, and demyelinating diseases is outlined in Table 1. All lab tests utilized the patient's blood.

**Table 1:** Autoimmune and infectious workup MRSA: Methicillin-resistant Staphylococcus aureus; RPR: Rapid plasma reagin; HIV: Human immunodeficiency virus; JCV: John Cunningham virus; ANA: Antinuclear antibody; ANCA: Antineutrophil cytoplasmic antibodies

Lab	Value	Normal Range
Cryptococcal antigen	Negative	Negative
Fungitell	< 31 pg/mL	< 60 pg/mL
Coccidioidies antibody	Negative	Negative
Aspergillus antigen	< 0.5	< 0.5
Histoplasma antigen	Not detected	Not detected
MRSA culture	Negative	Negative
RPR	Nonreactive	Nonreactive
HIV antibody and antigen	Nonreactive	Nonreactive
Karius	Negative	Negative
JCV DNA	< 500 copies/mL	< 500 copies/mL
C-reactive protein	30.8 mg/L	< 10.0 mg/L
Sedimentation rate	4 mm/hr	0 – 20 mm/hr
ANA	Negative	Negative
ANCA	< 0.2 U	< 0.4 U
Rheumatoid factor	< 10 IU/mL	< 14 IU/mL

His laboratory testing also included a comprehensive metabolic panel (CMP) and T-lymphocyte panel. His CMP was significant for decreased protein of 6.0 g/dL (normal range: 6.4-8.9 g/dL) and slightly elevated glucose of 117 g/dL (normal range: 65-100 g/dL). His T-lymphocyte profile was significant for decreased total lymphocytes of 11% (normal range: 18-45%) and an elevated CD4/CD8 ratio of 3.31 (normal range: 0.70-2.30). A lumbar puncture was not performed.

He then underwent a left stereotactic brain biopsy with intra-operative frozen pathology notable for glial cells with macrophages initially concerning for low to moderate cellular glioma. Additional immunohistochemical staining revealed positive P53 in the inclusions, a polyclonal plasma cell population with positive CD45 and CD138, and a mixed polyclonal population of kappa and lambda. The diagnosis was confirmed via JCV DNA in-situ hybridization (ISH) from the biopsied brain tissue. Quantitative JCV DNA performed on a sample of the patient's blood was detected at < 500 copies/mL of JCV DNA in the specimen.

**Figure 2:**
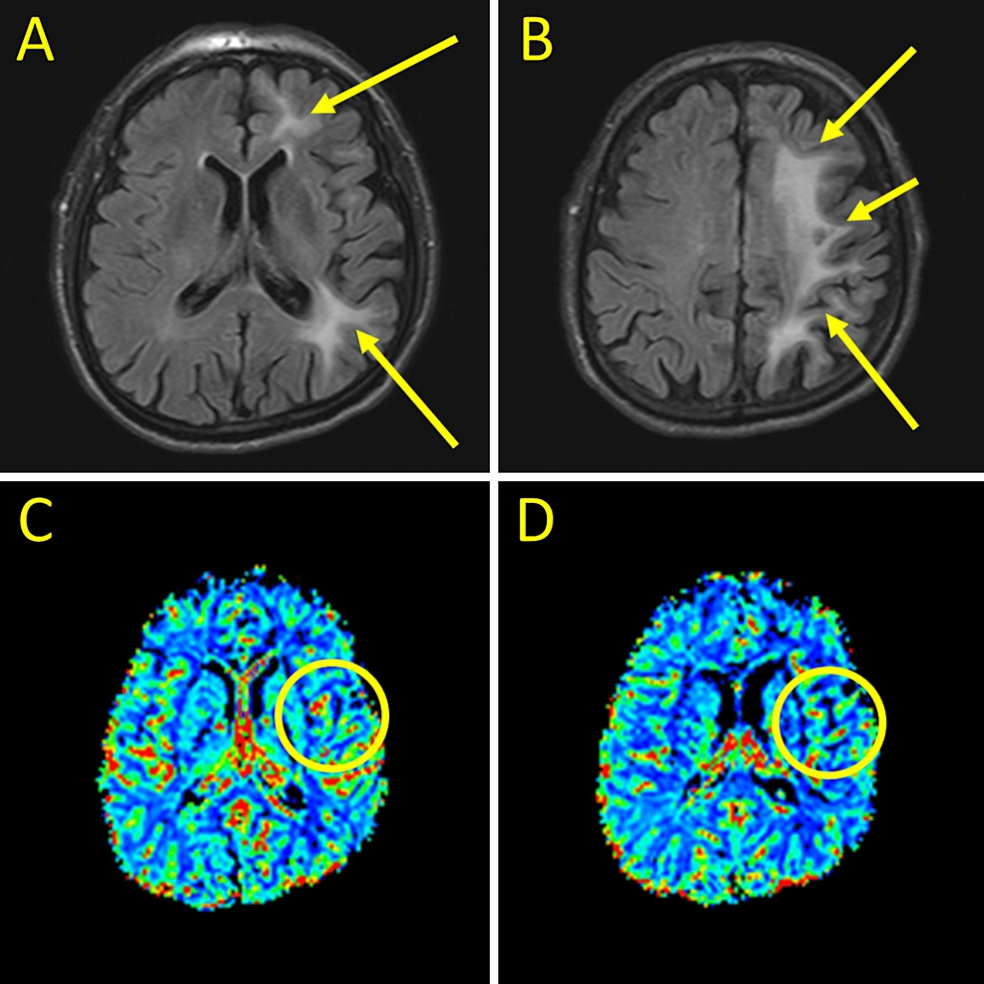
Key images from MRI brain with and without contrast and perfusion imaging (a) T2 flair post-contrast showing improvement of abnormal left-hemispheric signaling (yellow arrows). (b) T2 flair post-contrast showing abnormal left-hemispheric signaling, improved from initial study (yellow arrows). (c) MRI perfusion showing decreased cerebral blood volume compared to patient's initial study (yellow circle). (d) MRI perfusion showing persistence of decreased cerebral blood volume compared to prior study (yellow circle).

The patient was discharged without complication after a brain biopsy for outpatient management. Follow-up MRI brain perfusion five months later showed improved peripheral enhancement with no new foci of white matter enhancement (Figure 2).

Consideration for intervention included the administration of pembrolizumab or high-dose steroids. However, the patient began to improve spontaneously and no therapeutic medication was needed. He continues to improve with respect to memory, speech production, and to some degree, self-sufficiency.

## Discussion

Our review of the literature has shown that there are only 23 reported cases of PML in immunocompetent patients [2-11]. A previous review of the literature performed by Gheuens et al. yielded an additional 32 cases [9]. None of these cases initially presented as a primary CNS malignancy and, unlike our patient, 35 (64%) of these cases had significant underlying co-morbid conditions such as hepatitis B and C, liver cirrhosis, and end-stage renal disease on hemodialysis. In addition, 22 (40%) of cases did not have HIV serology available and 25 (45%) had PML diagnosis confirmed by brain biopsy.

Patients with PML often have medical histories with immunocompromising states, such as AIDS or malignancies [1,9]. In recent years, patients undergoing treatment with the alpha-4-beta-1 integrin inhibitor natalizumab have been noted to be the second largest group diagnosed with PML [12]. Less commonly, chronic diseases such as chronic kidney disease, liver disease, type 2 diabetes mellitus, and Alzheimer’s disease have been associated with PML diagnosis [1,2,9]. Physical exam findings associated with PML vary depending on the site of the lesion(s). Limb weakness, hemiparesis, ataxia, and gait disturbance are among the more common movement disorders associated with PML. Additional characteristics can include aphasia, vision deficits, and seizures. In rare and severe cases, brainstem involvement can also be seen [1]. MRI brain is typically characterized by hyperintense areas in T2-weighted images with diffuse, subcortical lesions located in white matter areas. MR spectroscopy is notable for increased choline and decreased NAA [13]. These findings are similarly seen in MR spectroscopy of primary CNS lymphoma [14]. Electroencephalography (EEG) findings have been previously described to show focal slowing early in the disease with later progression to diffuse abnormalities. In one example, a patient with natalizumab-associated PML was found to have severe dysfunction of the right hemisphere [15]. JCV DNA in CSF isolated by polymerase chain reaction (PCR) in combination with clinical and imaging findings confirms the diagnosis of PML [13]. Brain biopsy is considered to be the most reliable and accurate method for diagnosing PML, but it is no longer considered necessary in the modern day due to its invasive nature [1,12].

The patient described is not immunocompromised. MRI perfusion of the brain is of particular interest, as there was hyperperfusion that co-registered with affected areas. Due to the patient's immunocompetent state, the possibility of PML was not initially considered high on the differential diagnosis and tipped the scales toward biopsy. The combination of brain biopsy with characteristic features of a viral process plus positive JCV DNA ISH confirmed the diagnosis of PML. JCV quantitative DNA taken from the patient’s serum detected less than 500 copies per mL. This small number of viral copies could be explained by the patient’s competent immune system counteracting viral replication.

There are several limitations in our case report. First, this report describes a single case of PML in an immunocompetent patient, thus limiting the generalizability of our findings. Second, the lack of long-term follow-up does not allow us to exclude the possibility that the patient's symptoms could recur, regardless of his excellent short-term improvement. Third, we did not specifically perform CSF studies nor did we have the patient undergo extensive immunological or genetic testing to identify the potential reason why the JCV reactivated. Identifying the reason for JCV reactivation through a thorough review of immunological studies and whole exome sequencing is especially important when faced with PML in an immunocompetent patient, as it can lend insight into the mechanism of reactivation and potential novel treatment options [16]. Finally, previous cases of PML in immunocompetent patients offered the possibility that the patient was transiently immunodeficient at some point in their health history [2-5]. We cannot exclude that possibility in this case.

This case report demonstrates that abnormal neurological symptoms accompanied by increased perfusion and white matter expansion on MRI in an immunocompetent patient could be a rare presentation of PML. What started as a workup and treatment course for a malignant process changed completely once pathology indicated a viral process. Due to limited treatment options and lack of definitive evidence for efficacy, one may be skeptical of earlier diagnosis. The answer to this is that there may be modifiable factors with early discovery of PML that can lead to improvement of patient outcomes or change the course of an inappropriate intervention [1,2]. Our patient has spontaneously begun improving clinically with naming, repetition, and the ability to assist with self-care. He continues with a MRI imaging and outpatient follow-up every three months.

## Conclusions

PML is a progressive and often fatal disease more commonly found in immunocompromised hosts. While it is not impossible that an immunocompetent patient could become afflicted with the disease, this phenomenon is exceedingly rare. This case report describes one such patient who initially presented with abnormal neurological symptoms and MRI findings concerning for a malignancy. However, as revealed through careful and thorough workup, his diagnosis was confirmed to be PML. This discovery completely changed the course of his management and allowed for early and extensive monitoring of his disease course, most likely contributing to his excellent patient outcome. This report shows that when faced with abnormal neurologic findings and MRI imaging with increased perfusion and white matter expansion, the diagnosis of PML should not be excluded even when a patient is otherwise immunocompetent.
